# Supplementary material for: Investigation of locomotive syndrome improvement by total hip arthroplasty in patients with hip osteoarthritis: A before-after comparative study focusing on 25-question geriatric locomotive function scale
Source: PLoS One. 2025 Jun 9;20(6):e0315353. doi: 10.1371/journal.pone.0315353 (PMC12148190; doi:10.1371/journal.pone.0315353)
Supplement: S2 File — (DOCX) [file pone.0315353.s002.docx]

**S2 File. Codebook**

- Sex

1: Male
2: Female

- Diagnosis

1: Primary hip osteoarthritis
2: Secondary hip osteoarthritis

- THA

1: Unilateral
2: Bilateral

- Surgical procedure

1: AMIS approach
2: Transgluteal approach

- JOA staging (non-operated side)

0: Stage 0

1: Stage 1
2: Stage 2

3: Stage 3

4: Stage 4

5: After THA

- JOA staging (operated side)

0: Stage 0

1: Stage 1
2: Stage 2

3: Stage 3

4: Stage 4

- Before THA

Stand-up test: CDL stage

0: No locomotive syndrome
1: Stage 1
2: Stage 2
3: Stage 3

- Before THA

Stand-up test: Score

0: Score 0

1: Score 1
2: Score 2
3: Score 3
4: Score 4

5: Score 5

6: Score 6

7: Score 7

8: Score 8

- Before THA

Two-step test: CDL stage

0: No locomotive syndrome
1: Stage 1
2: Stage 2
3: Stage 3

- Before THA

GLFS-25: CDL stage

0: No locomotive syndrome
1: Stage 1
2: Stage 2
3: Stage 3

- Before THA

Total CDL stage

0: No locomotive syndrome
1: Stage 1
2: Stage 2
3: Stage 3

- 3 months after THA

Stand-up test: CDL stage

0: No locomotive syndrome
1: Stage 1
2: Stage 2
3: Stage 3

- 3 months after THA

Stand-up test: Score

0: Score 0

1: Score 1
2: Score 2
3: Score 3
4: Score 4

5: Score 5

6: Score 6

7: Score 7

8: Score 8

- 3 months after THA

Two-step test: CDL stage

0: No locomotive syndrome
1: Stage 1
2: Stage 2
3: Stage 3

- 3 months after THA

GLFS-25: CDL stage

0: No locomotive syndrome
1: Stage 1
2: Stage 2
3: Stage 3

- 3 months after THA

Total CDL stage

0: No locomotive syndrome
1: Stage 1
2: Stage 2
3: Stage 3

- Changes in the total CDL

-1: One-stage deterioration

0: No change
1: One-stage improvement
2: Two-stage improvement
3: Three-stage improvement

- Improvements in the total CDL

0: Non-improvement
1: Improvement

- Before THA

GLFS-25: Q1-4

0: No pain
1: Mild pain
2: Moderate pain

3: Considerable pain

4: Severe pain

- Before THA

GLFS-25: Q5-14, Q16-21

0: Not difficult
1: Mildly difficult
2: Moderately difficult

3: Considerably difficult

4: Extremely difficult

- Before THA

GLFS-25: Q15

0: More than 2-3 km
1: Approximately 1 km
2: Approximately 300 m

3: Approximately 100 m

4: Approximately 10 m

- Before THA

GLFS-25: Q22, 23

0: Not restricted
1: Slightly restricted
2: Restricted about half the time

3: Considerably restricted

4: Gave up all activities

- Before THA

GLFS-25: Q24, 25

0: Have not felt anxious
1: Have occasionally felt anxious
2: Have sometimes felt anxious

3: Have often felt anxious

4: Have constantly felt anxious

- 3 months after THA

GLFS-25: Q1-4

0: No pain
1: Mild pain
2: Moderate pain

3: Considerable pain

4: Severe pain

- 3 months after THA

GLFS-25: Q5-14, Q16-21

0: Not difficult
1: Mildly difficult
2: Moderately difficult

3: Considerably difficult

4: Extremely difficult

- 3 months after THA

GLFS-25: Q15

0: More than 2-3 km
1: Approximately 1 km
2: Approximately 300 m

3: Approximately 100 m

4: Approximately 10 m

- 3 months after THA

GLFS-25: Q22, 23

0: Not restricted
1: Slightly restricted
2: Restricted about half the time

3: Considerably restricted

4: Gave up all activities

- 3 months after THA

GLFS-25: Q24, 25

0: Have not felt anxious
1: Have occasionally felt anxious
2: Have sometimes felt anxious

3: Have often felt anxious

4: Have constantly felt anxious

- Improvement

GLFS-25: Q1-25

0: Non-improvement
1: Improvement
