# Supplementary material for: Investigation of locomotive syndrome improvement by total hip arthroplasty in patients with hip osteoarthritis: A before-after comparative study focusing on 25-question geriatric locomotive function scale
Source: PLoS One. 2025 Jun 9;20(6):e0315353. doi: 10.1371/journal.pone.0315353 (PMC12148190; doi:10.1371/journal.pone.0315353)
Supplement: S3 File — (DOCX) [file pone.0315353.s003.docx]

**S3 File.** **STROBE Statement—checklist of items that should be included in reports of observational studies**

|  | Item No. | Recommendation | Page  No. | Relevant text from manuscript |
| --- | --- | --- | --- | --- |
| **Title and abstract** | 1 | (*a*) Indicate the study’s design with a commonly used term in the title or the abstract | Page 1 | Line 1-4 |
|  |  | (*b*) Provide in the abstract an informative and balanced summary of what was done and what was found | Page 3-4 | Line 47-70 |
| Introduction | | | |  |
| Background/rationale | 2 | Explain the scientific background and rationale for the investigation being reported | Page 4-5 | Line 72-106 |
| Objectives | 3 | State specific objectives, including any prespecified hypotheses | Page 5 | Line 106-109 |
| Methods | | | |  |
| Study design | 4 | Present key elements of study design early in the paper | Page 6 | Line 113 |
| Setting | 5 | Describe the setting, locations, and relevant dates, including periods of recruitment, exposure, follow-up, and data collection | Page 6, 7 | Line 117-118, 147-152 |
| Participants | 6 | (*a*) Give the eligibility criteria, and the sources and methods of selection of participants. Describe methods of follow-up | Page 6 | Line 131-134 |
|  |  | (*b*) For matched studies, give matching criteria and number of exposed and unexposed |  | Not applicable |
| Variables | 7 | Clearly define all outcomes, exposures, predictors, potential confounders, and effect modifiers. Give diagnostic criteria, if applicable | Page 7-8 | Line 154-158 |
| Data sources/ measurement | 8* | For each variable of interest, give sources of data and details of methods of assessment (measurement). Describe comparability of assessment methods if there is more than one group | Page 8-9 | Line 160-190 |
| Bias | 9 | Describe any efforts to address potential sources of bias | Page 8 | Line 168-171, 176-180 |
| Study size | 10 | Explain how the study size was arrived at | Page 9 | Line 199-203 |

Continued on next page

| Quantitative variables | 11 | Explain how quantitative variables were handled in the analyses. If applicable, describe which groupings were chosen and why | Page 9 | Line 194-198 |
| --- | --- | --- | --- | --- |
| Statistical methods | 12 | (*a*) Describe all statistical methods, including those used to control for confounding | Page 9-10 | Line 192-198, 204-205 |
|  |  | (*b*) Describe any methods used to examine subgroups and interactions |  | Not applicable |
|  |  | (*c*) Explain how missing data were addressed | Page 6 | Line 133-134 (Figure 1) |
|  |  | (*d*) If applicable, explain how loss to follow-up was addressed | Page 6 | Line 133-134 (Figure 1) |
|  |  | (*e*) Describe any sensitivity analyses |  | Not applicable |
| Results | | | | |
| Participants | 13* | (a) Report numbers of individuals at each stage of study—eg numbers potentially eligible, examined for eligibility, confirmed eligible, included in the study, completing follow-up, and analysed | Page 6-7 | Line 130-139 |
|  |  | (b) Give reasons for non-participation at each stage | Page 6 | Line 133-134 |
|  |  | (c) Consider use of a flow diagram | Page 6-7 | Line 135-136 (Figure 1) |
| Descriptive data | 14* | (a) Give characteristics of study participants (eg demographic, clinical, social) and information on exposures and potential confounders | Page 10 | Line 208-211 (Table 1) |
|  |  | (b) Indicate number of participants with missing data for each variable of interest | Page 6-7 | Line 133-136 (Figure 1) |
|  |  | (c) Summarise follow-up time (eg, average and total amount) | Page 6 | Line 117-118, 131-133 |
| Outcome data | 15* | Report numbers of outcome events or summary measures over time |  | Not applicable |
| Main results | 16 | (*a*) Give unadjusted estimates and, if applicable, confounder-adjusted estimates and their precision (eg, 95% confidence interval). Make clear which confounders were adjusted for and why they were included | Page 10-21 | Line 216-270 |
|  |  | (*b*) Report category boundaries when continuous variables were categorized |  | Not applicable |
|  |  | (*c*) If relevant, consider translating estimates of relative risk into absolute risk for a meaningful time period |  | Not applicable |

Continued on next page

| Other analyses | 17 | Report other analyses done—eg analyses of subgroups and interactions, and sensitivity analyses |  | Not applicable |
| --- | --- | --- | --- | --- |
| Discussion | | | | |
| Key results | 18 | Summarise key results with reference to study objectives | Page 22 | Line 271-282 |
| Limitations | 19 | Discuss limitations of the study, taking into account sources of potential bias or imprecision. Discuss both direction and magnitude of any potential bias | Page 25 | Line 347-351 |
| Interpretation | 20 | Give a cautious overall interpretation of results considering objectives, limitations, multiplicity of analyses, results from similar studies, and other relevant evidence | Page 22-25 | Line 283-346 |
| Generalisability | 21 | Discuss the generalisability (external validity) of the study results | Page 25 | Line 354-362 |
| Other information | |  | | |
| Funding | 22 | Give the source of funding and the role of the funders for the present study and, if applicable, for the original study on which the present article is based | Page 26 | Line 369-373 |

*Give information separately for cases and controls in case-control studies and, if applicable, for exposed and unexposed groups in cohort and cross-sectional studies.

**Note:** An Explanation and Elaboration article discusses each checklist item and gives methodological background and published examples of transparent reporting. The STROBE checklist is best used in conjunction with this article (freely available on the Web sites of PLoS Medicine at http://www.plosmedicine.org/, Annals of Internal Medicine at http://www.annals.org/, and Epidemiology at http://www.epidem.com/). Information on the STROBE Initiative is available at www.strobe-statement.org.
