# Supplementary material for: Investigation of locomotive syndrome improvement by total hip arthroplasty in patients with hip osteoarthritis: A before-after comparative study focusing on 25-question geriatric locomotive function scale
Source: PLoS One. 2025 Jun 9;20(6):e0315353. doi: 10.1371/journal.pone.0315353 (PMC12148190; doi:10.1371/journal.pone.0315353)
Supplement: S4 File — (PDF) [file pone.0315353.s004.pdf]

S4 File. 25-Question Geriatric Locomotive Function Scale

Over the past month, have you experienced any pain or had difficulties with activities of daily living?
Please answer the following 25 questions to help determine your risk of locomotive syndrome.

☐ **Following are questions about your body pain for the last one month:**						
Q1	Did you have any pain (including numbness) in your neck or upper limbs (shoulder, arm, or hand)?	No pain	Mild pain	Moderate pain	Considerable pain	Severe pain
Q2	Did you have any pain in your back lower back or buttocks?	No pain	Mild pain	Moderate pain	Considerable pain	Severe pain
Q3	Did you have any pain (including numbness) in your lower limbs (hip, thigh, knee, calf, shin, ankle, or foot)?	No pain	Mild pain	Moderate pain	Considerable pain	Severe pain
Q4	To what extent has it been painful to move your body in daily life?	No pain	Mild pain	Moderate pain	Considerable pain	Severe pain
☐ **Following are questions about your usual daily life for the last one month:**						
Q5	To what extent has it been difficult to get up from a bed or lie down?	Not difficult	Mildly difficult	Moderately difficult	Considerably difficult	Extremely difficult
Q6	To what extent has it been difficult to stand up from a chair?	Not difficult	Mildly difficult	Moderately difficult	Considerably difficult	Extremely difficult
Q7	To what extent has it been difficult to walk inside the house?	Not difficult	Mildly difficult	Moderately difficult	Considerably difficult	Extremely difficult
Q8	To what extent has it been difficult to put on and take off shirts?	Not difficult	Mildly difficult	Moderately difficult	Considerably difficult	Extremely difficult
Q9	To what extent has it been difficult to put on and take off trousers and pants?	Not difficult	Mildly difficult	Moderately difficult	Considerably difficult	Extremely difficult
Q10	To what extent has it been difficult to use the toilet?	Not difficult	Mildly difficult	Moderately difficult	Considerably difficult	Extremely difficult
Q11	To what extent has it been difficult to wash your body in the bath?	Not difficult	Mildly difficult	Moderately difficult	Considerably difficult	Extremely difficult
Q12	To what extent has it been difficult to go up and down stairs?	Not difficult	Mildly difficult	Moderately difficult	Considerably difficult	Extremely difficult
Q13	To what extent has it been difficult to walk briskly?	Not difficult	Mildly difficult	Moderately difficult	Considerably difficult	Extremely difficult
Q14	To what extent has it been difficult to keep yourself neat?	Not difficult	Mildly difficult	Moderately difficult	Considerably difficult	Extremely difficult
Q15	How far can you keep walking without rest? (please select the closet answer)	More than 2-3 km	approximately 1 km	approximately 300 m	approximately 100 m	approximately 10 m
Q16	To what extent has it been difficult to go out to visit neighbors?	Not difficult	Mildly difficult	Moderately difficult	Considerably difficult	Extremely difficult
Q17	To what extent has it been difficult to carry objects weighing approximately 2 kilograms (2 standard milk bottles or 2 PET bottles each containing 1 liter)?	Not difficult	Mildly difficult	Moderately difficult	Considerably difficult	Extremely difficult
Q18	To what extent has it been difficult to go out using public transportation?	Not difficult	Mildly difficult	Moderately difficult	Considerably difficult	Extremely difficult
Q19	To what extent have simple tasks and housework (preparing meals, cleaning up, etc.) been difficult?	Not difficult	Mildly difficult	Moderately difficult	Considerably difficult	Extremely difficult
Q20	To what extent have load-bearing tasks and housework (cleaning the yard, carrying heavy bedding, etc.) been difficult?	Not difficult	Mildly difficult	Moderately difficult	Considerably difficult	Extremely difficult

Q21	To what extent has it been difficult to perform sports activity (jogging, swimming, gate ball, dancing, etc.)?	Not difficult	Mildly difficult	Moderately difficult	Considerably difficult	Extremely difficult
Q22	Have you been restricted from meeting your friends?	Not restricted	Slightly restricted	Restricted about half the time	Considerably restricted	Gave up all activities
Q23	Have you been restricted from joining social activities (meeting friends, playing sport, engaging in activities and hobbies, etc.)?	Not restricted	Slightly restricted	Restricted about half the time	Considerably restricted	Gave up all activities
Q24	Have you ever felt anxious about falls in your house?	Have not felt anxious	Have occasionally felt anxious	Have sometimes felt anxious	Have often felt anxious	Have constantly felt anxious
Q25	Have you ever felt anxious about being unable to walk in the future?	Have not felt anxious	Have occasionally felt anxious	Have sometimes felt anxious	Have often felt anxious	Have constantly felt anxious
**Enter the number of answers** →		0 points =	1 points =	2 points =	3 points =	4 points =
**Add up the number of points** →		**Total**		**points**		
