# Supplementary material for: Investigation of locomotive syndrome improvement by total hip arthroplasty in patients with hip osteoarthritis: A before-after comparative study focusing on 25-question geriatric locomotive function scale
Source: PLoS One. 2025 Jun 9;20(6):e0315353. doi: 10.1371/journal.pone.0315353 (PMC12148190; doi:10.1371/journal.pone.0315353)
Supplement: Attachment — Submitted filename: Peer Review - Locomotive.docx [file pone.0315353.s005.docx]

**Peer Review – Luqman Abass**

Investigation of locomotive syndrome improvement by total hip arthroplasty in patients with hip osteoarthritis: a before-after comparative study focusing on 25-question geriatric locomotive function scale

**Originality and Novelty**

The study presents original research investigating the effectiveness of locomotive syndrome (LS) improvement three months after total hip arthroplasty (THA) in patients with hip osteoarthritis (OA). The use of the total clinical decision limits (CDL) 3-stage scale to identify specific trends in LS improvement based on the 25-Question Geriatric Locomotive Function Scale (GLFS-25) is a novel approach that adds value to the existing literature.

**Methodology and Technical Standards**

The study employs a before-after comparative design with a sample size of 273 patients, which is adequate for the research question. The methodology is well-described, including patient selection criteria, outcome measures, and statistical analyses. The use of standardized tests (stand-up test, two-step test, and GLFS-25) follows established guidelines from the Japanese Orthopedic Association, ensuring high technical standards.

**Data Analysis and Presentation**

The statistical analyses, including the Wilcoxon signed-rank test and Paired t-test, are appropriate for the study design and data types. The results are presented clearly in tables and figures, providing a comprehensive overview of the findings. The scatter plot (Fig 2) effectively illustrates the relationship between pre-THA GLFS-25 scores and score changes post-THA.

**Conclusions and Data Support**

The conclusions drawn from the study are presented appropriately and are well-supported by the data. The finding that approximately half of the patients showed improvement in LS three months after THA, with significant improvements in locomotion movements, is clearly demonstrated by the results.

**Language and Presentation**

The manuscript is written in standard English and presented in an intelligible fashion. The structure follows a typical scientific paper format, with clear sections for introduction, methods, results, and discussion.

**Ethical Standards**

The study adheres to ethical standards, with approval obtained from the Research Ethics Committee of the University of Miyazaki, School of Medicine (Approval No. O-0783). The researchers have appropriately addressed the ethical considerations for both retrospective and prospective data collection, including the use of an opt-out method for retrospective data.

**Reporting Guidelines and Data Availability**

The manuscript appears to adhere to appropriate reporting guidelines for observational studies. However, a statement regarding data availability should be included to ensure compliance with community standards.

**Recommendations**

1. Include a clear statement on data availability.

2. Consider discussing the limitations of the study, such as the potential impact of polyarticular disease and previous arthroplasties on the results.

3. Elaborate on the clinical implications of the findings, particularly regarding rehabilitation strategies post-THA.

4. Provide more context on how these results compare to existing literature on LS improvement after THA.
